# A novel solid self-nanoemulsifying drug delivery system (S-SNEDDS) for improved stability and oral bioavailability of an oily drug, 1-palmitoyl-2-linoleoyl-3-acetyl-rac-glycerol

**DOI:** 10.1080/10717544.2017.1344335

**Published:** 2017-07-04

**Authors:** Kyeong Soo Kim, Eun Su Yang, Dong Shik Kim, Dong Wuk Kim, Hye Hyun Yoo, Chul Soon Yong, Yu Seok Youn, Kyung Taek Oh, Jun-Pil Jee, Jong Oh Kim, Sung Giu Jin, Han Gon Choi

**Affiliations:** a College of Pharmacy & Institute of Pharmaceutical Science and Technology, Hanyang University, Sangnok-gu, Ansan, South Korea;; b College of Pharmacy, Yeungnam University, Gyongsan, South Korea;; c School of Pharmacy, Sungkyunkwan University, Jangan-gu, Suwon, South Korea;; d College of Pharmacy, Chung-Ang University, Seoul, South Korea;; e College of Pharmacy, Chosun University, Gwangju, South Korea;; f Department of Pharmaceutical Engineering, Dankook University, Cheonan, South Korea

**Keywords:** Oily drug, 1-palmitoyl-2-linoleoyl-3-acetyl-rac-glycerol, S-SNEDDS, hydroxypropyl methylcellulose, calcium silicate, solubility, bioavailability

## Abstract

To develop a novel solid self-nanoemulsifying drug delivery system (S-SNEDDS) for a water-insoluble oily drug, 1-palmitoyl-2-linoleoyl-3-acetyl-rac-glycerol (PLAG) with improved stability and oral bioavailability, numerous S-SNEDDS were prepared with surfactant, hydrophilic polymer, antioxidant, and calcium silicate (porous carrier) using the spray-drying method. Their physicochemical properties were evaluated using emulsion droplet size analysis, SEM and PXRD. Moreover, the solubility, dissolution, stability, and pharmacokinetics of the selected S-SNEDDS were assessed compared with the drug and a commercial soft capsule. Sodium lauryl sulfate (SLS) and hydroxypropyl methylcellulose (HPMC) with the highest drug solubility were selected as surfactant and hydrophilic polymer, respectively. Among the antioxidants tested, only butylated hydroxyanisole (BHA) could completely protect the drug from oxidative degradation. The S-SNEDDS composed of PLAG/SLS/HPMC/BHA/calcium silicate at a weight ratio of 1: 0.25: 0.1: 0.0002: 0.5 provided an emulsion droplet size of less than 300 nm. In this S-SNEDDS, the drug and other ingredients might exist in the pores of carrier and attach onto its surface. It considerably improved the drug stability (about 100 vs. 70%, 60 °C for 5 d) and dissolution (about 80 vs. 20% in 60 min) compared to the commercial soft capsule. Moreover, the S-SNEDDS gave higher AUC, C_max_, and T_max_ values than the commercial soft capsule; in particular, the former improved the oral bioavailability of PLAG by about 3-fold. Our results suggested that this S-SNEDDS provided excellent stability and oral bioavailability of PLAG. Thus, this S-SNEDDS would be recommended as a powerful oral drug delivery system for an oily drug, PLAG.

## Introduction

1-Palmitoyl-2-linoleoyl-3-acetyl-rac-glycerol (PLAG, Supplementary Figure S1) is a chemically synthesized monoacetyldiglyceride, which is extracted from the antlers of sika deer. PLAG prevents chemotherapy-induced neutropenia by modulating neutrophil transmigration (Shin et al., 2014; Yoon et al., 2015). This drug is an effective agent for the treatment of the growth of metastatic biliary cancer (Kim et al., 2009). However, PLAG is an oily drug, which is synthesized by reacting glycerol, palmitic acid, and linoleic acid (Lee et al., 2014). Moreover, it has a low bioavailability due to its poor water solubility and rapid decomposition (Yoo et al., 2016; Ryu et al., 2017). Therefore, to overcome this problem, the drug has been commercially developed as a soft capsule (Rockpid^®^; Enzychem Co., Seoul, South Korea) which contains 300 mg PLAG. However, the oral bioavailability of such a conventional soft capsule is still unsatisfactory, because an oily drug hardly dissolves in the gastrointestinal tract due to its high lipophilicity, even though it might permeate well through the body membranes. There has been not reported on any formulation of PLAG except soft capsule. Moreover, until now, there has been no information on the conversion of an oily drug to a practical solid pharmaceutical product.

Thus, in our research, to develop a novel PLAG-loaded solid self-nanoemulsifying drug delivery system (S-SNEDDS) with improved stability and oral bioavailability, numerous S-SNEDDS were prepared with surfactant, hydrophilic polymer, antioxidant, and porous carrier using the spray-drying method. Their physicochemical properties were evaluated using droplet size analysis, scanning electron microscopy (SEM), and powder X-ray diffraction (PXRD). Moreover, the solubility, dissolution, stability, and pharmacokinetics of selected S-SNEDDS were assessed compared with the drug and a commercial soft capsule.

A liquid self-nanoemulsifying drug delivery system (SNEDDS), an isotropic mixture of oils and surfactants, can speedily produce oil/water nanoemulsions upon gentle agitation and achieve great solubility and bioavailability (Seo et al., 2013, 2015a). Similarly, an S-SNEDDS, a solid form of liquid SNEDDS, can form oil-in-water nanoemulsions (usually less than 300 nm in droplet size) in the gastrointestinal tract (Kim et al., 2014; Rashid et al., 2015b). This system is a promising drug delivery system for the most improved solubility and oral bioavailability among solubility-enhancing techniques (Seo et al., 2015a). Furthermore, it has been frequently developed since it has the advantage of both a liquid SNEDDS and solid dosage form. Namely, this system can produce a large interfacial surface area for increasing drug solubility and oral bioavailability, and has simplified industrial manufacture, reduced production cost and improved stability as well as better patient compliance (Seo et al., 2015b).

## Materials and methods

### Materials

PLAG (99.7%), the commercial soft capsule (Rockpid^®^) and PLAG-d3 (1-palmitoyl-2-linoleoyl-3-acetyl-d3-rac-glycerol; internal standard for *in vivo* assay) were kindly provided by Enzychem Co. (Seoul, South Korea). Sorbitan monooleate 80 (Span^®^ 80), polysorbate 80 (Tween^®^ 80), sodium lauryl sulfate (SLS), ascorbic acid, butylated hydroxyanisole (BHA), butylated hydroxytoluene (BHT), sodium sulfite (Na_2_SO_3_), sodium bisulfite (NaHSO_3_), sodium metabisulfite (Na_2_S_2_O_5_), ethylenediaminetetraacetic acid (EDTA), dl-α-tocopherol and calcium silicate were obtained from Daejung Chemical Co. (Siheung, South Korea). Poloxamer^®^ 188, poloxamer^®^ 407, polyvinylpyrrolidone (PVP^®^ K30), Cremophor^®^ EL and Cremophor^®^ RH40 were purchased from BASF (Ludwigshafen, Germany). Sodium carboxymethyl cellulose (sodium CMC), hydroxypropyl methylcellulose (HPMC 4000 & 15000) and hydroxypropyl cellulose (HPC-L^®^) were provided by Shin-Etsu Co. (Tokyo, Japan). Dextran, polyvinyl alcohol (PVA), ascorbic acid, BHT, EDTA, dl-α-tocopherol, and BHA were obtained from Sigma-Aldrich Co. (St. Louis, MO). Capryol^®^ 90, Labrasol^®^, Labrafil^®^ M 2125CS, Lauroglycol^TM^ 90 and Transcutol^®^ P were obtained from Gattefossé (Saint-Priest, France). Carbopol^®^ was obtained from Lubrizol (Cleveland, OH). Hydroxypropyl-β-cyclodextrin (HP-β-CD) and β-cyclodextrin (β-CD) were purchased from Roquette (Lestrem, France). All other chemicals and solvents were of reagent grade and were used without additional purification.

### Solubility determination

An excess quantity of PLAG liquid (about 200 mg) was added to 10 mL of 1% aqueous solution containing 1% surfactant, and/or 1% polymer. These solutions were vortexed, shaken in a water bath at 25 °C for 7 d for reach the equilibrium state of PLSA solubility and centrifuged at 10,000 *g* for 15 min (Kim et al., 2016a; Yousaf et al., 2016b). Each middle layer was carefully taken, and serially diluted with the mobile phase. The PLAG concentrations were measured by an HPLC system (1220; Agilent, Santa Clara, CA). A Zorbax Eclipse XDB column (Agilent, 4.6 mm i.d. × 250 mm, 5 μm; Santa Clara, CA) was used. The mobile phase consisted of acetonitrile and isopropanol (55 : 45, v/v). The column temperature was 30 °C, the flow rate was 1 mL/min and the eluent was monitored at a wavelength of 210 nm. Samples were dissolved in mobile phase. After filtration (0.45 μm), aliquots of 20 μL were injected and eluted with the mobile phase under the above chromatographic conditions. This HPLC method was validated for linearity over the concentration range of 0.7813–100 μg/mL (*r*^2^ = .9999).

Moreover, various PLAG-loaded S-SNEDDS formulations were added to 50 mL of distilled water, and their drug solubility was determined, as described above.

### Oxidative degradation of PLAG

Aliquots of PLAG (10 mg) and various antioxidants (0.07 mg) were added to 10 mL of 0.1% H_2_O_2_/0.05% Tween 80 solution, respectively. Additionally, 10 mg PLAG and 0.001–0.07 mg BHA were added to 10 mL of the above H_2_O_2_ solution, respectively. These solutions were kept at the accelerated conditions of 40 °C for 4 d, and their drug concentrations were checked, as described above.

### Preparation of S-SNEDDS

Based on the solubility test, SLS and HPMC 15000 (abbreviated as ‘HPMC’) were selected as surfactant and hydrophilic polymer, respectively. PLAG (1 g) and various amounts of SLS, HPMC, and BHA were dissolved in 300 mL of 10% ethanol solution. Then, 0.5 g of calcium silicate was suspended in these resulting solutions, and spray-dried using a lab-scale mini spray-dryer (Büchi B-290; Büchi Co., Flawil, Switzerland) under the following drying conditions: inlet temperature = 60 °C, outlet temperature = 30 °C, feeding flow rate = 5 mL/min, air pressure = 4 kg/cm^2^, and aspirator pressure = −25 mbar.

### Morphological and physical characterization

#### Emulsion droplet size

Each S-SNEDDS (100 mg) was dispersed with 10 mL distilled water by vortex mixing (30 s). Afterwards, its emulsion droplet size was analyzed by dynamic light scattering using a Zetasizer Nano ZS (Malvern Instruments, Worcestershire, United Kingdom) at 633 nm (wavelength) and 90° (scattering angle) at 25 °C. The z-average diameter and polydispersity index (PDI) of the emulsions were derived from cumulated data by Automeasure software (Malvern Instruments, Worcestershire, United Kingdom).

#### Scanning electron microscope (SEM)

The morphologies of S-SNEDDS and ingredients were examined using an SEM (S-4800; Hitachi, Tokyo, Japan). Each sample was mounted on a brass stub using tape to secure the sample and platinum-coated by an EMI Tech Ion Sputter (K575K) at 15 mA for 240 s.

#### Powder X-ray diffraction (PXRD)

The crystalline properties of S-SNEDDS and ingredients were analyzed using a PXRD spectrometer (D/MAS-2500 PC; Rigaku, Tokyo, Japan). The samples were analyzed using Cu-Kα radiation generated at 40 mA and 40 kV. The data were obtained from a 2θ angle range of 10–60° using a step width of 0.02 °C at ambient temperature.

### *In vitro* drug release

Drug release tests were performed with S-SNEDDS, the drug and the commercial soft capsule containing 300 mg PLAG employing USP dissolution testing apparatus II (paddle method; Hanson, Chatsworth, CA) at 37 ± 0.5 °C at a speed of 100 rpm with 900 mL of dissolution medium. A pH 6.8 buffer solution containing 2.5% SLS and distilled water was used as the dissolution medium. Samples (1 mL) were withdrawn at 5, 10, 15, 30, 45, and 60 min, and then the PLAG concentrations were analyzed using the HPLC method, as given above.

### Stability

To investigate the stability of PLAG in the S-SNEDDS, drug and commercial soft capsule, they were kept under forced degradation conditions of 60 °C for 5 d, and then their drug content was analyzed using the HPLC method, as seen above. Additionally, for thermodynamic stability of S-SNEDDS, S-SNEDDS was subjected to heating-cooling test (45 and 4 °C) and freeze thaw cycle (−21 and 25 °C) for 48 h. For centrifugation stress, the S-SNEDDS (100 mg) was dispersed with 10 mL distilled water and centrifuged at 3500 rpm for 15 min and observed for any phase separation (Singh et al., 2013).

### Oral bioavailability

The protocol of animal experimentation was performed according to NIH Policy and the Animal Welfare Act under the approval of the Institutional Animal Care and Use Committee (IACUC) at Hanyang University. Male Sprague Dawley rats (6–8 weeks of age, 280 ± 20 g) bought from Nara Biotech (Seoul, South Korea) had unrestricted access to usual laboratory food and drinking water under controlled conditions of 23–24 °C/50–60% RH before the study. A cannula was inserted into the right femoral artery with polyethylene tubing filled with heparin solution (50 IU/mL). The S-SNEDDS filled in a small capsule (#9; Suheung Capsule Co., Seoul, South Korea), content of the commercial soft capsule or drug equivalent to 200 mg/kg of PLAG were orally administered to the rats, respectively. Blood samples were collected from the right femoral artery after 0.05, 0.125, 0.25, 0.5, 1, 2, 3, 4, 6, and 8 h. Plasma samples (300 μL) were stored at −80 °C until analysis. Twenty microliters of 1% formic acid and 5 μL of 2% EDTA were added to each blood sample, vortexed for 5 s and centrifuged at 12,000 *g* for 5 min. The supernatant plasma (80 μL) was added to 100 μL of isopropanol and 1 mL of ethyl acetate/hexane (2/1, v/v) containing PLAG-d3 as an internal standard (1 ng/mL). This solution was vortexed for 10 min and centrifuged at 12,000 *g* for 5 min. The upper liquid layer (900 μL) was then collected and evaporated via N_2_ gas. Subsequently, the sample was reconstituted in 150 μL of acetonitrile/isopropanol (2/1, v/v), vortexed for 5 min, and centrifuged at 12,000 *g* for 5 min. The supernatant (5 μL) was injected into the LC–MS/MS apparatus. In this assay, the plasma concentrations of PLAG were assessed using a Triple Stage Quadrupole (TSQ) Quantum Access MAX (Thermo Scientific, San Jose, CA) with an electrospray ionization (ESI) interface which was employed in the positive ion mode ([M + H]^+^). The drug assay was performed on a Phenomenex Kinetex C18 column (2.1 mm i.d. × 50 mm, 2.6 μm; Torrance, CA) maintained at 40 °C. The mobile phase consisted of solutions A and B (6 : 4, volume ratio); solutions A and B were composed of 0.1% formic acid in 10 mM ammonium formate/acetonitrile and 0.1% formic acid in 10 mM ammonium formate/isopropanol at a volume ratio of 1 : 9, respectively. The flow rate of the mobile phase was 0.3 mL/min. The ESI-MS data were acquired in the positive mode and the conditions of MS analysis were as follows: spray voltage, 4500 V; vaporizer temperature, 350 °C; sheath gas pressure, 60 psi; aux gas pressure, 20 psi; capillary temperature, 350 °C. The analytics were detected by monitoring the transitions *m*/*z* 652.7 → 379.2 and 655.7 → 382.2 for PLAG and internal standard (PLAG-d3), respectively. The calibration curve of the concentration range 1–1000 ng/mL was obtained. Quantification was performed by multiple reaction monitoring (MRM) of the protonated precursor ions and the related product ions, using the ratio of the area under the peak for each solution and a weighting factor of 1/*y*^2^. The calibration curve was plotted over a range of 1 ∼ 1000 ng/mL (*r*^2^ = .9999) in plasma. The lower limit of quantification of PLAG was 1 ng/mL. In the validation process, intra- and inter-day differences were determined and the differences were shown to be within an acceptable range (Ryu et al., 2017).

## Results and discussion

### Preparation and physicochemical characterization

PLAG is an oily drug with very little solubility in water, resulting in low stability and oral bioavailability. In this study, in order to overcome these problems of oily PLAG, a S-SNEDDS, a solid dosage form, was developed with a surfactant, hydrophilic polymer, antioxidant, and carrier using a spray-drying technique. In general, liquid SNEDDS, nanoemulsion systems, have been prepared with surfactant and oil (Rashid et al., 2015a). However, the oil is not necessary in the preparation of PLAG-loaded SNEDDS, because PLAG is an oily drug. In this development, a hydrophilic polymer is needed to make the S-SNEDDS maintain a more sturdy solid state. In addition, hydrophilic polymers play a role as a precipitation inhibitor to form supersaturable SNEDDS (Chen et al., 2012; Oh et al., 2014; Mustapha et al., 2016) and polymeric micelle**-**forming agents (Thapa et al., 2016; Truong et al., 2016).

First, in order to choose a surfactant suitable for the S-SNEDDS of PLAG, a solubility test was performed with 1% surfactant aqueous solution. Among the surfactants investigated for the equilibrium solubility study, the highest solubility of PLAG was observed in SLS (2.1 ± 0.3 mg/mL); poloxamer 407 and Tween 80 showed the second highest drug solubility (Supplementary Figure S2(A)). Thus, SLS, a solid surfactant was selected for further study. When the large amount of SLS, an anionic surfactant was orally administered, it can irritate the gastric mucosa and possibly lead to gastrointestinal adverse events. However, its small amounts have been frequently used in the development of oral pharmaceutical products due to its approval by FDA (Liu et al., 2016; Kim et al., 2016b; Morcos et al., 2017). For choosing an appropriate hydrophilic polymer, the effect of the polymer on drug solubility in 1% SLS aqueous solution was investigated (Supplementary Figure S2(B)). Instead of distilled water, 1% SLS aqueous solution was used as a solubility medium, since PLAG is not miscible in distilled water. The drug solubility of cellulose derivatives, such as HPC-L, HPMC 4000, and HPMC 15000, was significantly higher than those of other polymers. In particular, HPMC 15000 provided the highest solubility of PLAG (4.7 ± 0.2 mg/mL). Hence, HPMC 15000 (abbreviated as ‘HPMC’) was chosen for further study.

**Figure 1.**
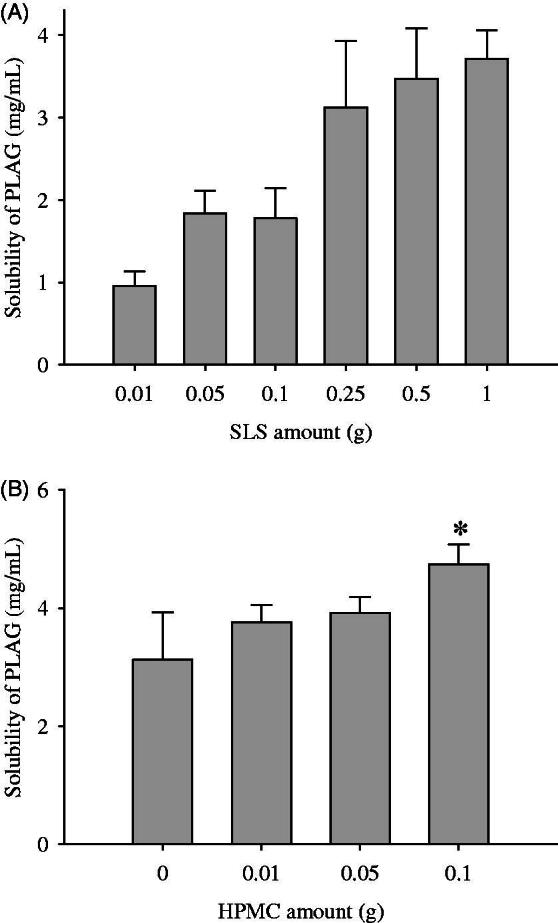
Effect of carriers on the drug solubility: (A) SLS; (B) HPMC. The drug (1 g), various amounts (0.01–2 g) of SLS and/or various amounts (0.01–1 g) of HPMC were dissolved in 60 mL of 10% ethanol solution, respectively, and then spray-dried. Each value represents the mean ± SD (*n* = 3).

To select the optimal amounts of ingredients, 1 g drug, various amounts of SLS (0.01–1 g) and HPMC were dissolved in 90% ethanol solution and spray-dried, followed by investigation of their drug solubility (Figure 1). The effect of SLS amount on the drug solubility was checked (Figure 1(A)). As the amounts of SLS were increased to 0.25 g, the drug solubility was increased (Yousaf et al., 2015a; Kim et al., 2016b; Yousaf et al., 2016b). However, the formulations prepared with more than 0.25 g SLS gave no significant increase in drug solubility (3.13 ± 0.80 vs. 3.47 ± 0.61 vs. 3.72 ± 0.34 mg/mL). SLS causes a toxic effect at a high oral dose (Lee et al., 2014; Yousaf et al., 2016a). Thus, in respect of high drug solubility and low SLS amounts, the amount of SLS was selected to be 0.25 g.

Subsequently, to choose an appropriate amount of HPMC, formulations were prepared with various amounts of HPMC (0.01–0.1 g), fixing the amounts of drug (1 g) and SLS (0.25 g), and the solubility of the drug was checked (Figure 1(B)). In this preparation, more than 0.1 g HPMC was not soluble in 300 mL 10% ethanol; therefore, a formulation with more than 0.1 g HPMC could not be prepared. The greater the increase in HPMC amounts, the better the drug solubility. Hence, the HPMC amount was fixed at 0.1 g.

**Figure 2.**
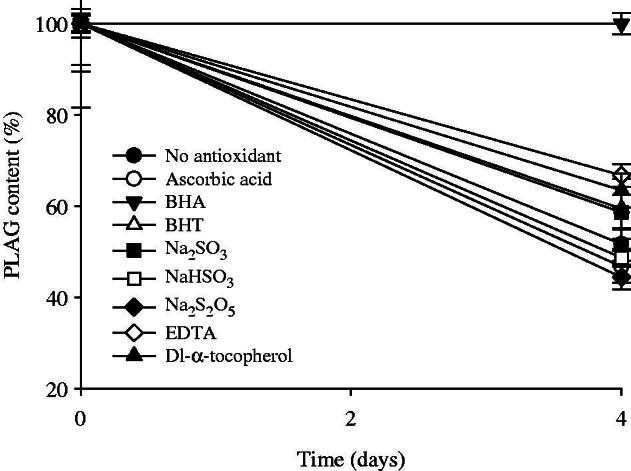
Effect of antioxidants on the stability of drug in 0.1% H_2_O_2_ solution at the accelerated conditions of 40 °C for 4 d. Each value represents the mean ± SD (*n* = 3).

PLAG is unstable under oxidative conditions due to its oily nature (Sherry et al., 2013). To select an appropriate antioxidant for excellent stability of PLAG, the effect of antioxidants on the oxidative degradation of the drug was investigated (Figure 2). Ascorbic acid, BHA, BHT, Na_2_SO_3_, NaHSO_3_, Na_2_S_2_O_5_, EDTA, and dl-α-tocopherol were used as the antioxidants. When no antioxidant was added, PLAG was degraded to about 52% in the accelerated conditions of 40 °C for 4 d. Among the antioxidants, BHA, EDTA, and dl-α-tocopherol significantly improved the oxidative stability of the drug, but the others did not. In particular, BHA could completely protect PLAG from oxidative degradation at 40 °C for at least 4 d (99.9 ± 2.3 vs. 51.7 ± 3.6%). BHA has been used as a potential phenolic antioxidant in foods as well as in pharmaceutical products (Carocho & Ferreira, 2013). Afterwards, to determine the amounts of BHA, 0.001–0.07% BHA against drug was added to the H_2_O_2_ solution at the same accelerated conditions, and the drug concentrations were investigated. The amounts of BHA at 0.01, 0.02, 0.05, 0.1, 0.4, and 0.7% BHA were 94.1 ± 4.2, 96.1 ± 3.2, 96.2 ± 2.8, 96.9 ± 4.2, 96.6 ± 2.5, and 99.9 ± 2.3%, respectively. Thus, the addition of BHA increased the oxidative stability of BHA in the accelerated conditions. The BHA amounts above 0.02% gave more than 96% of the drug concentration. Even though they were not significantly different, 0.01% BHA amounts did not. Moreover, the American Food and Drug Administration (FDA) permits BHA to a maximum concentration of 0.02% (Pérez Martín et al., 2014). Therefore, 0.02% BHA against drug was the concentration chosen to be added as a drug stabilizer.

On the other hand, the solid dosage form for oily drugs is useful due to its ease of production, high chemical stability, and good patient compliance. In general, S-SNEDDS are prepared by spray-drying liquid SNEDDS with a porous carrier. In this study, calcium silicate was employed as the porous carrier (Saluja et al., 2015; Weerapol et al., 2015; Dening et al., 2016). It has been reported to provide good flow ability, large surface area and good tableting properties (Hentzschel et al., 2012).

PLAG (1 g), 0.25 g SLS, 0.1 g HPMC, 0.0002 g BHA, and 0.5 g calcium silicate were dissolved or suspended in 90% ethanol solution (300 mL) and spray-dried, leading to production of the PLAG-loaded S-SNEDDS. This S-SNEDDS provided an emulsion droplet size of about 270 nm (PDI = 0.252 ± 0.006) (raw data not shown), suggesting its excellent self-emulsifying capacity (Oh et al., 2011; Kim et al., 2013). Therefore, this S-SNEDDS formulation composed of 300 mg PLAG, 75 mg SLS, 30 mg HPMC, 0.06 mg BHA, and 150 mg calcium silicate was chosen for further study.

**Figure 3.**
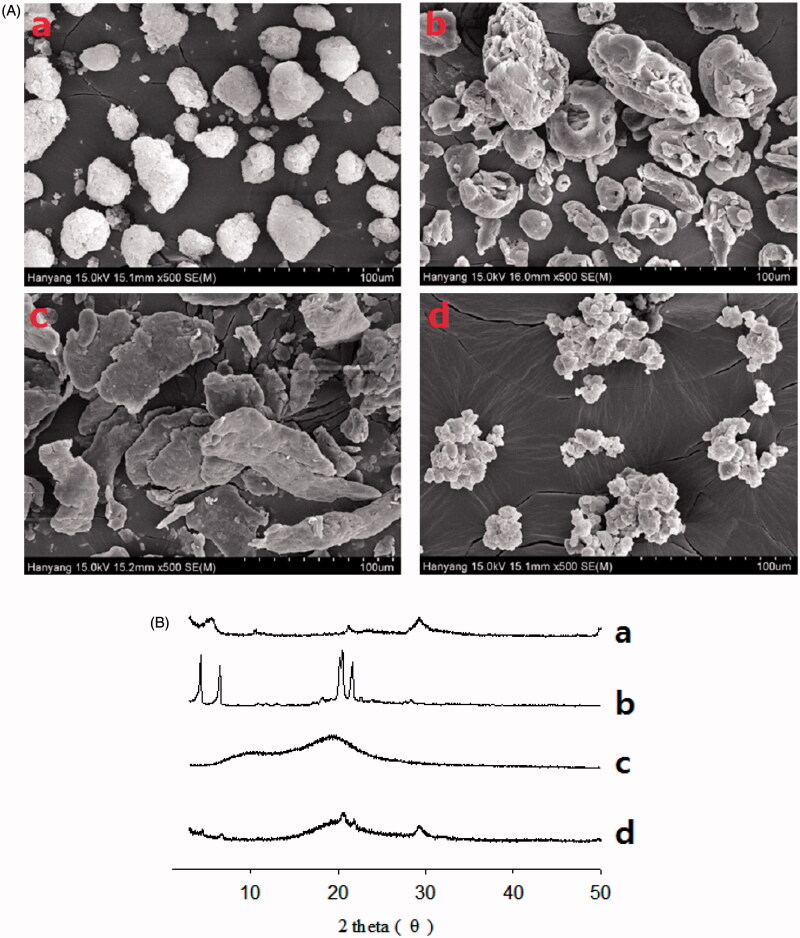
Scanning electron micrographs (A) and PXRD patterns (B): (a) calcium silicate; (b) SLS; (c) HPMC; (d) S-SNEDDS.

Figure 3(A,B) represent the scanning electron micrographs and PXRD patterns of the S-SNEDDS and ingredients, respectively. In this test, PLAG, an oily liquid form, could not be determined. Calcium silicate (Figure 3(A-a)), SLS (Figure 3(A-b)) and HPMC (Figure 3(A-c)) had an irregular porous shape, rough-surfaced crystals and flat-surfaced plate crystals, respectively (Yousaf et al., 2015b; Kim et al., 2016a). The S-SNEDDS (Figure 3(A-d)) showed an irregular rough-surfaced shape. In addition, the particle size of the S-SNEDDS was smaller than calcium silicate. Our results suggest that PLAG, SLS, and HPMC might exist in the pores of calcium silicate and attach onto its surface. Calcium silicate (Figure 3(B-a)) and SLS (Figure 3(B-b)) had a pattern consistent with an intrinsically crystalline nature (Kim et al., 2016b). HPMC (Figure 3(B-c)) showed relatively weak peaks, which were negligible and not distinctive (Yang et al., 2013; Kim et al., 2016d). Similarly, all the distinguishing crystalline peaks were shown in the S-SNEDDS (Figure 3(B-d)). Therefore, the S-SNEDDS scarcely influenced the crystallinity of the ingredients.

**Figure 4.**
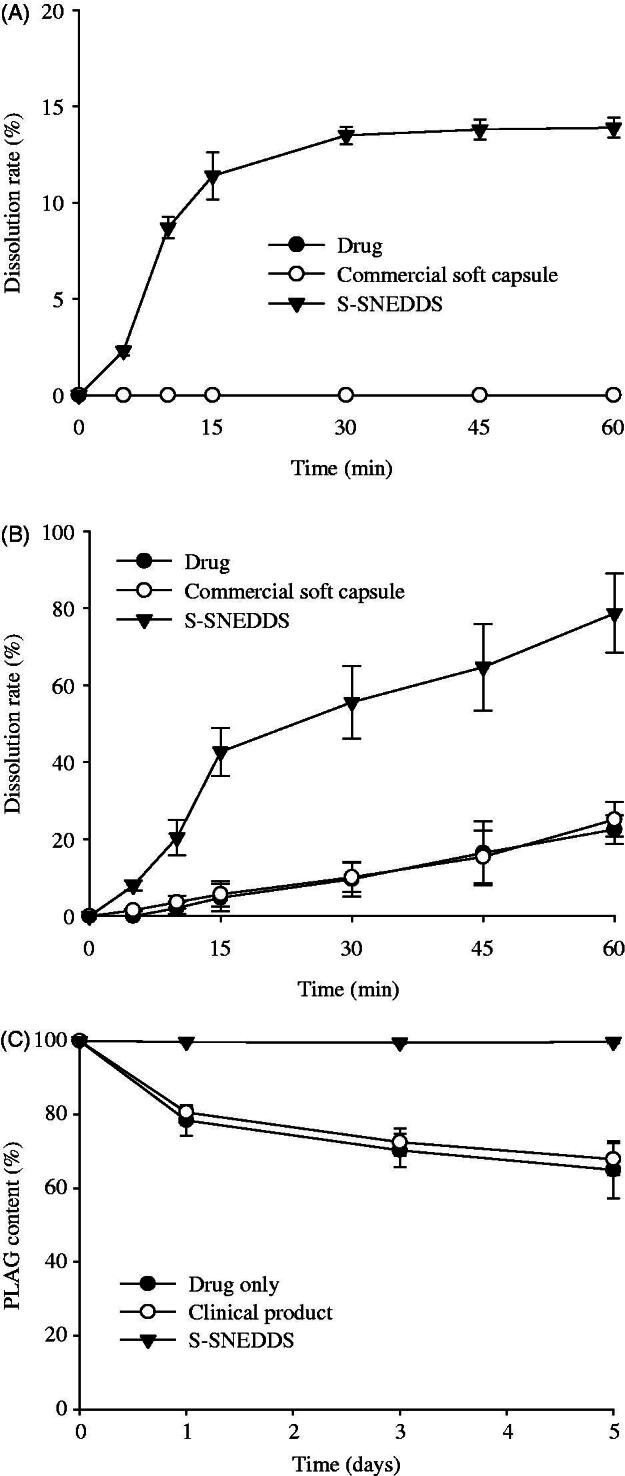
Dissolution profile of PLAG in distilled water (A), pH 6.8 buffer solution containing 2.5% SLS (B) and stability at 60 °C for 5 d (C) from S-SNEDDS, commercial soft capsule and drug. Each value represents the mean ± SD (*n* = 6).

The drug solubility and dissolution of the drug, commercial soft capsule, and S-SNEDDS were compared. The drug and commercial soft capsule hardly showed any solubility of PLAG. However, the S-SNEDDS improved the solubility of the drug (2.5 ± 0.3 mg/mL). Moreover, the S-SNEDDS significantly increased the dissolution of the drug compared to PLAG only and the commercial soft capsule in distilled water as well as in pH 6.8 buffer solution containing 2.5% SLS (Figure 4) (Kim et al., 2015; Kim et al., 2016c). The drug and commercial soft capsule hardly provided any drug dissolution in distilled water; however, the S-SNEDDS improved the dissolution (Figure 4(A)). In pH 6.8 buffer solution containing 2.5% SLS, the S-SNEDDS significantly enhanced dissolution of drug about four-fold compared to the others (about 80 vs. 20 vs. 20% at 60 min) (Figure 4(B)).

The comparable stability of drug was evaluated by the drug content at 60 °C for 5 d (Figure 4(C)) (Kim et al., 2015; Kim et al., 2016c). The drug and commercial soft capsule showed an obvious decrease in drug content (about 30%) over a period of 5 d. However, the S-SNEDDS showed no significant difference in drug content, suggesting that it considerably improves the stability of PLAG. The first-order degradation rate constant and shelf-life (t_90_) of S-SNEDDS were obtained as follows: Ln (C*_t_*/C_o_) = −*kt*, and *t*_90_ = 0.105/*k*, where C_o_ and C_t_ are the initial and remaining concentrations of PLAG at time *t,* respectively (Dadparvar et al., 2014). The degradation of S-SNEDDS provided first order kinetics of 0.001 h^−1^ (*r*^2^ = .9994) and shelf-life (*t*_90_) of 105 d at 60 °C (raw data not shown). Thermodynamic stability and self-nanoemulsification study are important for development of liquid SNEDDS (Inugala et al., 2015). S-SNEDDS passed the thermodynamic stability tests without any signs of phase separation during alternative temperature cycle, freeze thaw cycle, and centrifugation. Therefore, our S-SNEDDS and its nanoemulsion gave an excellent stability.

### Pharmacokinetics

**Figure 5.**
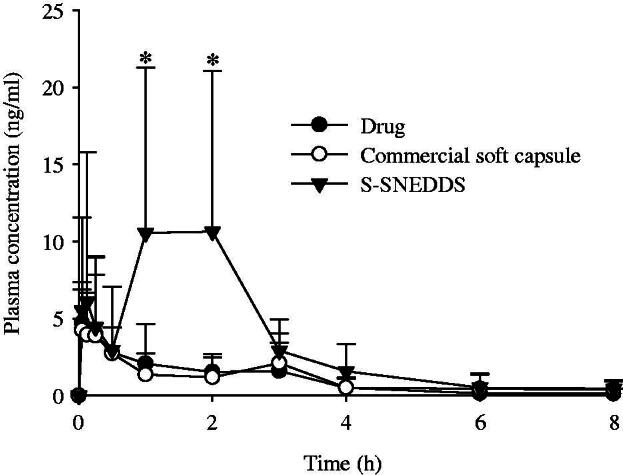
Mean plasma level–time profiles of PLAG after oral administration of S-SNEDDS, commercial soft capsule and drug at a dose equivalent to 200 mg/kg drug in rats. Each value designates the mean ± SD. (*n* = 8). The S-SNEDDS was composed of PLAG/SLS/HPMC/BHA/calcium silicate at a weight ratio of 1: 0.25: 0.1: 0.0002: 0.5. *p < .05 compared with drug only and commercial soft capsule.

Figure 5 demonstrates the plasma concentration profiles of drug after oral administration at a dose equivalent to 200 mg/kg drug in rats. The S-SNEDDS was composed of PLAG/SLS/HPMC/BHA/calcium silicate at a weight ratio of 1: 0.25: 0.1: 0.0002: 0.5. At all times, the plasma concentrations for the S-SNEDDS were higher than those for the drug and commercial soft capsule; in particular, they were significantly different at 1–2 h. Furthermore, it was observed that their profiles were different. The drug and commercial soft capsule gave a single peak. However, the S-SNEDDS showed a double peak at 0.25 and 1–2 h. It has been reported that lipid formulations, such as microemulsions (Larsen et al., 2013; Xiao et al., 2013), nanocrystal self-stabilized emulsions (Yi et al., 2016), and self-emulsifying pellets (Iosio et al., 2011) show the double peak plasma phenomenon due to enterohepatic circulation, efflux transporters and phase II conjugation, and lymphatic transport, respectively. Thus, in our study, such a double peak might be contributed by enterohepatic circulation and the complex absorption of PLAG involved in transport and metabolism (Chen et al., 2015). The pharmacokinetic parameters are summarized in Table 1. Critically, the S-SNEDDS gave higher AUC, C_max_, and T_max_ values than the drug and commercial soft capsule. In particular, the former greatly improved (3.4- and 3.2-fold, respectively), the AUC compared to the latter products. Our results suggest that the enhanced oral bioavailability of the S-SNEDDS is provided by improved drug solubility and dissolution (Kim et al., 2015, 2016d).

**Table 1.** Pharmacokinetic parameters.

Parameter	AUC (h·ng/mL)	C_max_ (ng/mL)	T_max_ (h)
Drug	8.39 ± 4.78	4.70 ± 4.46	0.60 ± 1.02
Commercial soft capsule	8.79 ± 5.32	4.25 ± 2.63	0.62 ± 1.01
S-SNEDDS	28.25 ± 24.25*	10.65 ± 10.41*	1.05 ± 0.69*

Each value represents the mean ± SD (*n* = 8).

The S-SNEDDS was composed of PLAG/SLS/HPMC/BHA/calcium silicate at a weight ratio of 1: 0.25: 0.1: 0.0002: 0.5.

* *p* < .05 compared with drug and commercial soft capsule.

## Conclusion

The S-SNEDDS composed of drug/SLS/HPMC/BHA/calcium silicate at a weight ratio of 1: 0.25: 0.1: 0.0002: 0.5 provided excellent solubility, stability, and oral bioavailability of PLAG. Thus, this S-SNEDDS would be recommended as a powerful oral drug delivery system for an oily drug, PLAG.

## Supplementary Material

IDRD_Choi_et_al_Supplemental_Content.docx
