# Supplementary material for: A novel solid self-nanoemulsifying drug delivery system (S-SNEDDS) for improved stability and oral bioavailability of an oily drug, 1-palmitoyl-2-linoleoyl-3-acetyl-rac-glycerol
Source: Drug Deliv. 2017 Jul 4;24(1):1018–25. doi: 10.1080/10717544.2017.1344335 (PMC8240999; doi:10.1080/10717544.2017.1344335)
Supplement: IDRD_Choi_et_al_Supplemental_Content.docx [file IDRD_A_1344335_SM0632.docx]

**Supplementary Information**

**A novel solid self-nanoemulsifying drug delivery system (S-SNEDDS) for improved stability and oral bioavailability of an oily drug, 1-palmitoyl-2-linoleoyl-3-acetyl-rac-glycerol**

Kyeong Soo Kim^1,^*, Eun Su Yang^1,^*, Dong Shik Kim^1^, Dong Wuk Kim^1^, Hye Hyun Yoo^1^, Chul Soon Yong ^2^, Yu Seok Youn^3^, Kyung Taek Oh^4^, Jun-Pil Jee^5^, Jong Oh Kim^2,‡^, Sung Giu Jin^1,6,‡^, Han-Gon Choi^1, ‡^

^1^College of Pharmacy & **Institute of Pharmaceutical Science and Technology,** Hanyang University, 55 Hanyangdaehak-ro, Sangnok-gu, Ansan 426-791, South Korea

^2^College of Pharmacy, Yeungnam University, 214-1, Dae-Dong, Gyongsan 712-749, South Korea

^3^School of Pharmacy, Sungkyunkwan University, 300 Cheoncheon-dong, Jangan-gu, Suwon 440-746, South Korea

^4^College of Pharmacy, Chung-Ang University, 221 Heuksuk-dong Dongjak-gu, Seoul 156-756, South Korea

^5^College of Pharmacy, Chosun University, 309 Pilmun-daero, Gwangju 61452, South Korea

^6^Department of Pharmaceutical Engineering, Dankook University, 119 Dandae-ro, Dongnam-gu, Cheonan, 330-714, South Korea

***** These authors contributed equally to this work.

^‡^Corresponding author: Prof. Han-Gon Choi

Tel.: +82-31-400-5802

Fax: +82-31-400-5958

E-mail: hangon@hanyang.ac.kr

^‡^Co-corresponding author: Prof. Sung Giu Jin

Tel: +82-41-550-3558

Fax: +82-41-550-3558

E-mail: sklover777@dankook.ac.kr

^‡^Co-corresponding author: Prof. Jong Oh Kim

Tel: +82-53-810-2813

Fax: +82-53-810-4654

E-mail: jongohkim@yu.ac.kr

**Fig. S1.** Structure of 1-palmitoyl-2-linoleoyl-3-acetyl-rac-glycerol (PLAG).

**Fig. S2.** Aqueous solubility of PLAG: (A) surfactant and (B) polymers in 1% SLS aqueous solution. Each value represents the mean ± S.D. (n = 3).

**Fig. S3.** Effect of antioxidants on the stability of drug in 0.1% H_2_O_2_ solution at the accelerated conditions of 40 °C for 4 days. Each value represents the mean ± S.D. (n = 3).
